# Case Report: SMART syndrome mimicking Todd’s paralysis after meningioma irradiation

**DOI:** 10.3389/fonc.2026.1917766

**Published:** 2026-08-19

**Authors:** Anneliese Troidle, Danzhu Zhao, Charles Rutter, Lisa Knopf, Narinder Maheshwari

**Affiliations:** 1 University of Connecticut School of Medicine, Farmington, CT, United States; 2 Department of Internal Medicine, University of Connecticut Health Center, Farmington, CT, United States; 3 Department of Radiation Oncology, Hartford Hospital, Hartford, CT, United States; 4 Department of Neurology, Hartford Hospital, Hartford, CT, United States

**Keywords:** meningioma, neurology, neuro-oncology, radiation oncology, SMART syndrome

## Abstract

Stroke-like Migraine Attacks after Radiation Therapy (SMART) syndrome is a rare, delayed complication of cerebral irradiation characterized by stroke-like symptoms, seizures, and/or headaches, with mean latency period of 11.2 years. It is most associated with gliomas and medulloblastomas and diagnosis is challenging due to symptom overlap with recurrent neurologic deficits. We present a 59-year-old male with history of right frontal convexity anaplastic meningioma complicated by seizure disorder and Todd’s paralysis, status post tumor resection, adjuvant radiation to 6000cGy, craniotomy and cranioplasty complicated by subdural abscess/empyema. Four years later, he presented with an epileptic seizure followed by progressive left facial and hand numbness with facial droop over 24 hours. Vitals, blood work, lumbar puncture and head computed tomography (CT) were unremarkable. CT angiography demonstrated asymmetric gyral enhancement and sulcal effacement within the right frontal lobe. Brain magnetic resonance imaging (MRI) demonstrated right hemispheric sulcal enhancement within the prior radiation field with cortical/leptomeningeal FLAIR hyperintensity and subtle diffusion restriction Electroencephalography revealed nonspecific right temporal and paracentral lateralized rhythmic discharges. Following multidisciplinary evaluation, the patient required a prolonged dexamethasone taper due to failed initial steroid taper and was initiated on amlodipine with continuation of levetiracetam, zonisamide, and clobazam. MRI at three months showed enhancement resolution with clinical improvement and no recurrent seizures. This case highlights SMART syndrome as a diagnosis of exclusion, notable for its shortened latency in comparison to the reported mean and rare association with meningioma, emphasizing the need for heightened clinical suspicion in patients with prior cerebral irradiation presenting with progressive neurologic deficits.

## Introduction

Stroke-like Migraine Attacks after Radiation Therapy (SMART) syndrome is a rare, delayed complication of cerebral irradiation that is characterized by a range of neurologic presentations including stroke-like symptoms, seizures, and headaches, with a mean latency period of approximately 11.2 years following radiation (1). It is most associated with gliomas and medulloblastomas and thought to involve dysfunction of cerebrovascular autoregulation mechanisms, neuronal dysfunction inducing trigeminovascular system impairment, cortical spreading depression, and seizures (1–3). Diagnosis is challenging in patients with prior history of neurological conditions with recrudescence of similar manifestations. We present a patient with a history of anaplastic meningioma status post irradiation therapy complicated by focal epilepsy and transient postictal deficits consistent with Todd’s paralysis. Four years later, he presented with a similar but significantly prolonged and persistent neurologic syndrome. Neuroimaging findings were consistent with SMART syndrome, and the deficits persisted ultimately requiring extended corticosteroid taper and calcium channel blocker therapy.

## Case presentation

Patient is a 59-year-old right-handed male with history of WHO grade 3 right frontal convexity anaplastic meningioma complicated by focal epilepsy and Todd’s paralysis status post tumor resection, adjuvant radiation to 6000cGy further complicated by subdural abscess/empyema status post cranioplasties. The patient received a 6MV dose painted VMAT 6000cGy to the operative bed with small margins and 58 Gray to operative bed with larger margins in 30 fractions at the right frontal convexity over 42 days. He presented to the emergency department four years later with a 10-minute episode of epileptic seizure despite levetiracetam and zonisamide compliance, followed by a prolonged episode of Todd’s paralysis of greater than 24 hours prompting urgent evaluation. The patient has previously experienced Todd’s paralysis after seizures and experienced similar clinical features to past episodes, though his symptoms reportedly would resolve within three to four hours. His vitals and blood work were largely unremarkable and physical exam revealed 3/5 strength in the left upper extremity and loss of the left nasolabial fold. Given these physical exam findings, National Institute of Health Stroke Scale (NIHSS) rating was 4. His head computed tomography (CT) was notable only for evidence of previously right cranioplasty. CT angiography (CTA) demonstrated asymmetric gyral enhancement and sulcal effacement within the right frontal lobe without concern for ischemic changes or moyamoya. Hyperemia noted on CTA thought to be of reactive etiology as opposed to a primary vascular etiology. T1 magnetic resonance imaging (MRI) with and without contrast showed right hemispheric sulcal enhancement in the region of prior radiation therapy and cortical/leptomeningeal FLAIR hyperintensity and enhancement with subtle diffusion restriction subjacent to the cranioplasty peripherally without concern for tumor recurrence, radiation necrosis, or prolonged post-ictal changes. Lumbar puncture revealed glucose and protein within normal range and did not reveal presence of white and red blood cells or xanthochromia, suggesting no microbial infection of the central nervous system and eliminating concern for leptomeningeal disease. Long term monitoring electroencephalography (EEG) revealed nonspecific right temporal and paracentral lateralized rhythmic discharges (LRDA).

## Timeline

**Figure 1:**
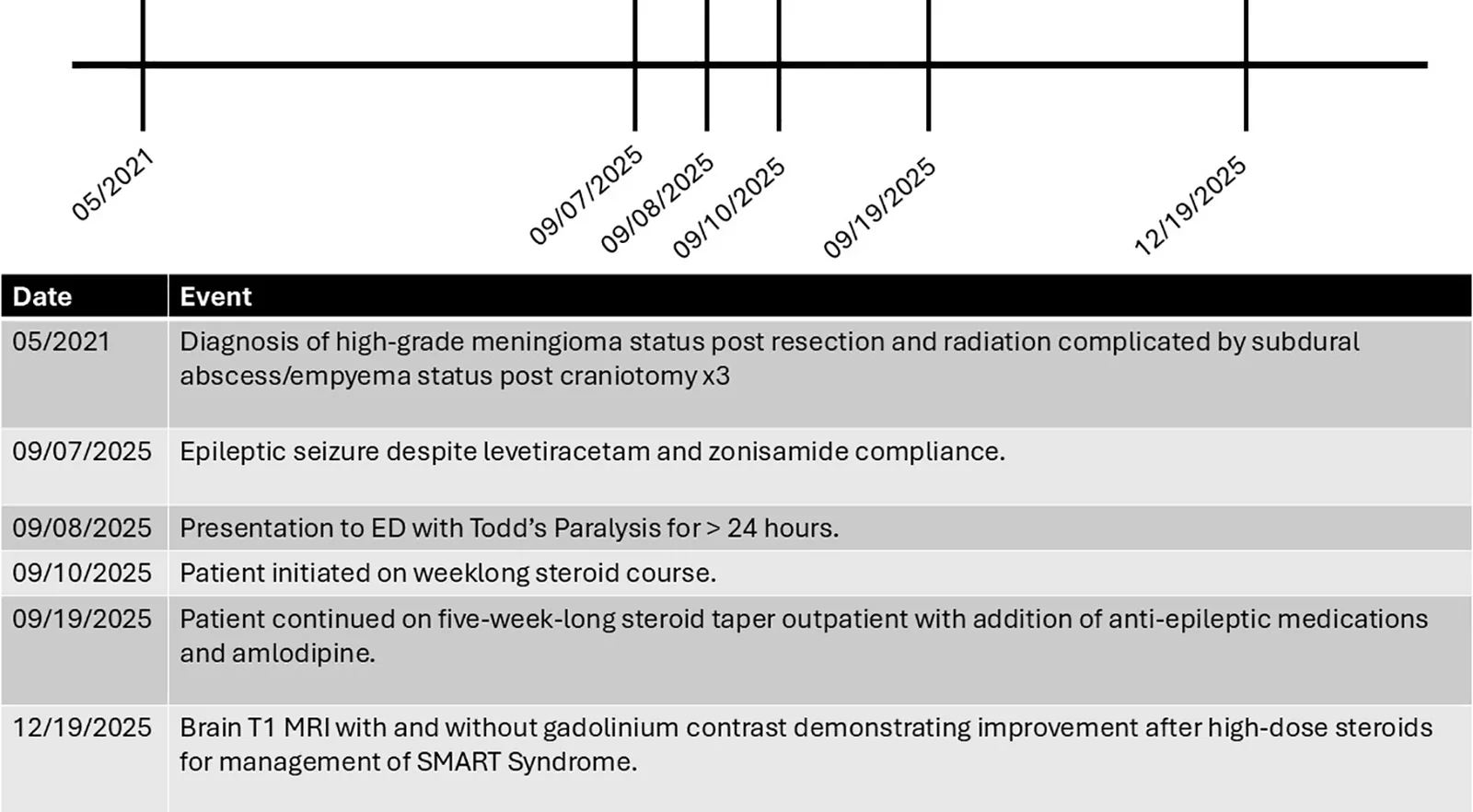
Timeline of the patient’s SMART syndrome course from initial radiation therapy to current clinical presentation.

Time course of the patient’s presentation is displayed in **Figure 1**.

## Diagnostic assessment and therapeutic intervention

**Figure 2:**
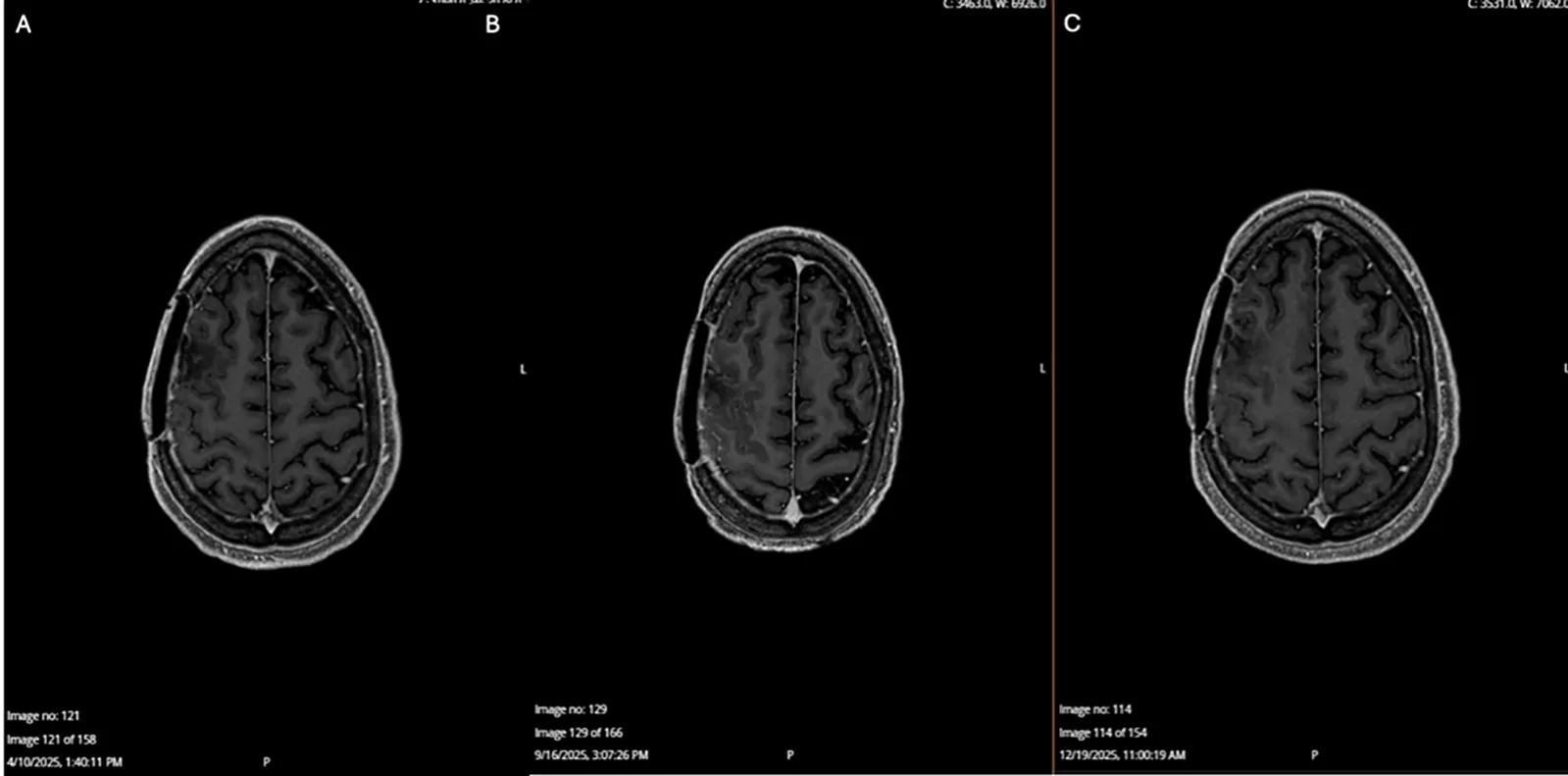
**(A–C)** Axial T1 MRI series with and without gadolinium contrast of the temporal progression of SMART syndrome changes from his baseline MRI with and without contrast before symptom onset that shows findings of right frontal craniectomy and cranioplasty **(A)**, the peak sulcal enhancement upon presentation on the right cortical/leptomeningeal FLAIR hyperintensity with associated enhancement beneath the cranioplasty peripherally **(B)**, and the rapid improvement with interval resolution of the cortical mantle diffusion restriction and pathologic superficial enhancement subjacent to the cranioplasty site after high dose steroids when re-imaged three months later **(C)**.

**Figure 3:**
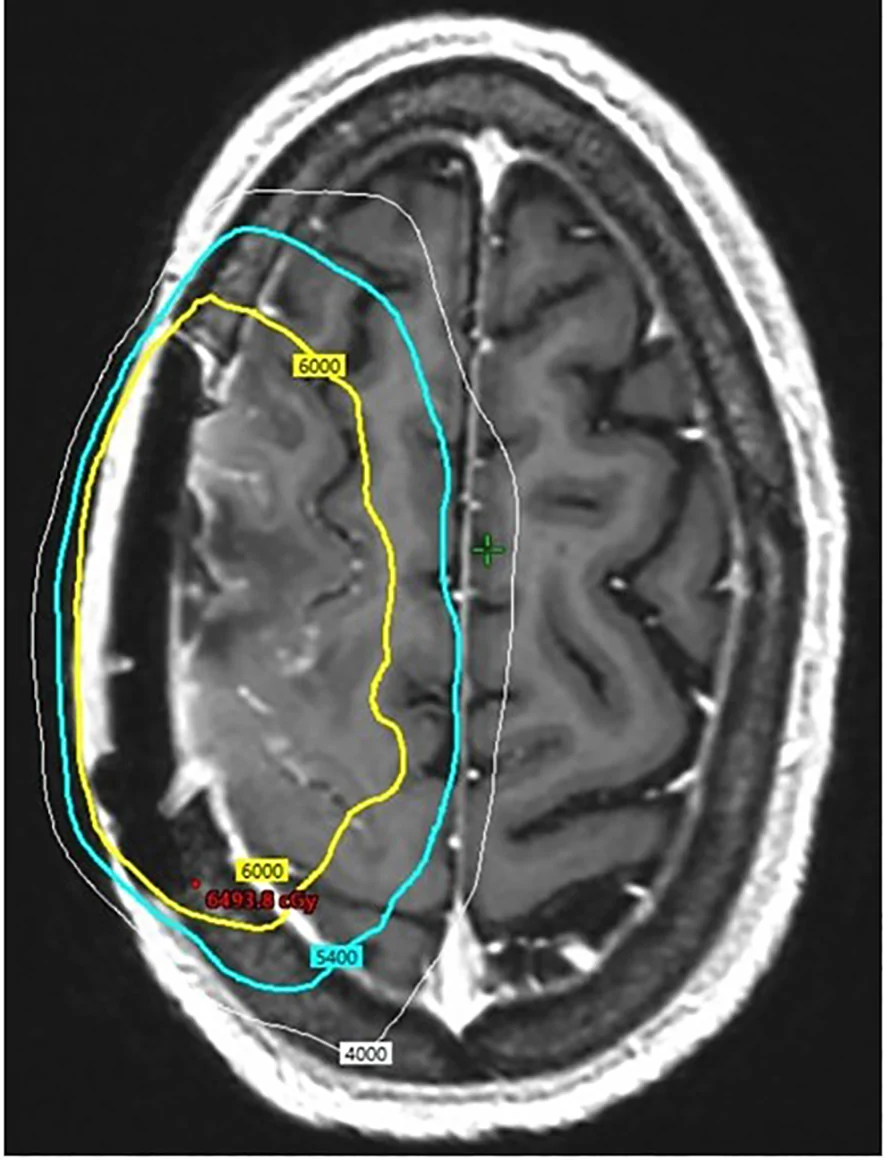
Dose distribution from the patient’s radiation therapy overlying the axial T1 MRI with and without gadolinium contrast image that captures the peak presentation of SMART syndrome, demonstrating the correlation between enhancement and dose. The patient received a 6 MV dose painted VMAT 6000 cGy to the operative bed with small margins and 58 Gy to the operative bed with large margins in 30 fractions at the right frontal convexity across 42 days.

The patient was discharged from the hospital with clobazam 10mg PO nightly for the LRDA noted on long-term monitoring (LTM) with EEG and was started on intravenous dexamethasone 10mg load with 4mg every six hours upon discharge for one week for suspected SMART syndrome. The patient followed up with radiation oncology following the completion of the dexamethasone course and reported notable but incomplete improvement in left upper extremity weakness and lack of further seizure activity. The patient was continued on a five-week dexamethasone taper course with addition of amlodipine 5mg daily and continuation of levetiracetam, zonisamide, and clobazam. Repeat MRI brain three months after the completion of the initial course of steroid treatment showed resolution of sulcal enhancement, suggesting that the steroid dose improved the inflammatory process rather than this patient’s presentation be the result of post-ictal changes as demonstrated in **Figures 2A–C** The patient had clinical improvement of left upper extremity strength and fine motor function without further seizure activity as shown in **Figure 3**.

## Discussion

SMART syndrome is a rare and diagnosis of exclusion that most commonly manifests decades after cranial irradiation, ranging from 1 to 35 years (4). Its clinical presentation frequently mimics more prevalent neurologic conditions, including cerebrovascular events, seizure-related phenomena, migraine disorders, and tumor recurrence, thereby posing diagnostic challenges. Diagnostic criteria initially proposed by Bartleson et al. and later updated in 2006 by Black et al. included: a prior history of cranial irradiation; prolonged yet reversible unilateral cortical signs and symptoms arising years after radiation exposure; transient, unilateral cortical gray matter enhancement on neuroimaging with relative sparing of white matter; and exclusion of alternative etiologies (4). Zheng et al. in 2015 proposed a similar diagnostic criterion, though highlights the need to exclude of recurrence of malignancy (5). Our patient met all criteria.

In the systematic review by Chow et al., SMART syndrome was most observed in patients with prior gliomas (28.6%), followed by medulloblastomas (25.0%), whereas only a small minority of cases were reported in association with meningiomas (3.6%) as seen in our patient (1). Lubotzky et al. detailed a case of a 10-year-old boy with previous metastatic group 3 medulloblastoma presented with similarly shorter than reported mean onset of SMART syndrome symptoms just two years after radiation therapy (6). Al Yasari et al. presented a case of an 18-year-old male with past history of craniopharyngioma for which he received photon therapy six years prior to symptom onset and diagnosis of SMART syndrome (7). Both presented with more typical SMART syndrome symptoms of headache and extremity weakness, though neither case reported the prolonged symptoms experienced by our patient, further highlighting the need to recognize how types of previously treated tumors may be associated with SMART syndrome and may require a heightened suspicion in tumors that are rarely associated with this syndrome.

The mechanisms underlying SMART syndrome remain incompletely understood. Proposed hypotheses include radiation-induced disruption of the blood–brain barrier and trigeminovascular system, potentially lowering the threshold for cortical spreading depression and thereby predisposing to seizures and migraine-like phenomena. These mechanisms share conceptual parallels with syndromes such as posterior reversible encephalopathy syndrome (PRES). Additionally, genetic susceptibility has been postulated, given phenotypic similarities to familial hemiplegic migraine, although definitive evidence remains limited (3, 4).

Management strategies for SMART syndrome remain largely supportive and symptom-directed, though there are currently no standardized guidelines. Antiseizure therapy is the most common empirical therapy, along with corticosteroids, though there are no controlled trials that have studied the efficacy of corticosteroid therapy (8). Prior literature reviews have suggested that clinical improvement may occur in the absence of corticosteroid therapy, raising questions regarding their role as standard of care (8). Calcium channel blockers have also been reported in select cases, potentially targeting cerebrovascular dysregulation similarly seen in PRES (9, 10). In this case, the patient demonstrated limited response to initial corticosteroid therapy, ultimately requiring a prolonged corticosteroid taper and adjunctive calcium channel blocker therapy. Although neurologic deficits incompletely resolved, follow-up neuroimaging revealed resolution of cortical enhancement, suggesting radiographic improvement. As previously discussed in Jia et al, SMART syndrome has been shown to spontaneously resolve, and though clinical improvement with administration of corticosteroid therapy is notable, it is not proof of efficacy nor therapeutically diagnostic of SMART syndrome (6). In a multicenter retrospective review, 45% of patients with diagnosed SMART syndrome were found to have recurrent episodes, and presence of ictal cortical FLAIR hyperintensity and longer episode duration were linked to lower risk of recurrence (11).

## Conclusion

This case is notable for its significantly shortened latency when compared to reported means and rare association with meningioma and highlights the need for a heightened index of suspicion in patients with prior cerebral irradiation who present with exacerbation of prior neurologic deficits rather than new symptoms. Larger studies are necessary to define the heterogeneity of neurologic presentations and to determine whether prior neurologic symptoms influence the likelihood of recurrence versus new symptom development. Further studies are also needed to clarify treatment guidelines and long-term efficacy.

## Data Availability

The original contributions presented in the study are included in the article/supplementary material. Further inquiries can be directed to the corresponding author.
